# Otub1 stabilizes MDMX and promotes its proapoptotic function at the mitochondria

**DOI:** 10.18632/oncotarget.14278

**Published:** 2016-12-27

**Authors:** Yingxiao Chen, Yue-Gang Wang, Yuhuang Li, Xiao-Xin Sun, Mu-Shui Dai

**Affiliations:** ^1^ Department of Molecular and Medical Genetics, School of Medicine, and The OHSU Knight Cancer Institute, Oregon Health and Science University, Portland, OR 97239, USA

**Keywords:** deubiquitinating enzymes, MDMX, Otub1, p53, apoptosis

## Abstract

Otub1 regulates p53 stability and activity via non-canonical inhibition of UbcH5, the MDM2 cognate ubiquitin-conjugating enzyme (E2). However, whether Otub1 regulates MDMX stability and activity is not clear. Here we report that Otub1 also suppresses MDM2-mediated MDMX ubiquitination in cells and *in vitro*, independently of its deubiquitinating enzyme activity. Consequently, overexpression of Otub1 markedly stabilized MDMX and increased its levels, whereas knockdown of Otub1 reduced the levels of MDMX. Interestingly, MDMX induced by Otub1 can localize to mitochondria in addition to the cytosol, enhance p53 phosphorylation at S46 (p53S46P) and promote mitochondria-mediated apoptotic pathway. Knockdown of MDMX reduced Otub1-induced p53S46P, which was shown to be critical for p53's mitochondrial function and apoptotic activity. Furthermore, Otub1 promotes UV-irradiation-induced p53S46P and apoptosis, which can be significantly inhibited by MDMX depletion. Together, these results suggest that Otub1 stabilizes MDMX and promotes p53S46P and mitochondria-mediated apoptosis, providing an alternative mechanism of Otub1's role in apoptosis.

## INTRODUCTION

The tumor suppressor protein p53 inhibits cell growth and proliferation in response to stress by transcriptionally activating or suppressing myriad target genes, whose protein products induce cell cycle arrest, cell death, senescence and other outcomes [1–3]. p53 can also directly trigger mitochondria-mediated apoptosis and necrosis through transcription-independent mechanisms [4–8]. p53 directly interacts with anti-apoptotic Bcl-2 family proteins such as Bcl-xL and Bcl-2 at mitochondria to neutralize their inhibition of proapoptotic proteins, leading to Bax or Bak oligomerization [7]. p53 can directly interact with Bax, leading to Bax oligomerization, mitochondria outer membrane permeabilization (MOMP) and cytochrome C release [4]. It can also bind to Bak and disrupt the Bak-MCL1 interaction, causing the oligomerization of Bak and release of cytochrome C as well [9]. A recent study showed that p53 also triggers mitochondria-dependent necrosis in response to oxidative stress by binding to the mitochondrial permeability transition pores (PTPs) [8]. Thus the cytoplasmic p53 plays a key role in negatively regulating cell growth by inducing cell death pathways.

Under physiological conditions, p53 is tightly controlled at low levels mainly by MDM2, a RING-finger domain-containing ubiquitin (Ub) ligase (E3) [10, 11] that mediates p53 ubiquitination and its subsequent proteasome degradation [12–14]. MDM2 also directly suppresses p53 activity by binding to and concealing the N-terminal transactivation domain of p53 [15, 16]. As MDM2 itself is a transcriptional target of p53, they form an auto-regulatory feedback loop [17–19], ensuring a tight control of homeostatic levels of both proteins. Various stress signals invoke diverse mechanisms to barricade the MDM2-mediated p53 suppression to stabilize and activate p53. The MDM2 homolog MDMX (also called MDM4) is also critical for the proper control of p53 levels and activity [20, 21]. Like MDM2, MDMX binds to p53 transactivation domain and suppresses its activity [22, 23]. Unlike MDM2, MDMX does not possess ubiquitin E3 activity towards p53 [24, 25]. Yet, it assists MDM2 to inhibit p53 by binding to MDM2 through their RING domains and stabilizes MDM2 [26–31], whereas MDM2 in turn ubiquitinates and degrades MDMX in response to DNA damage [32, 33]. Thus, the p53-MDM2-MDMX axis is critical for normal control of the levels and activity of p53 and is tightly regulated by various posttranslational modifications in response to stress.

Deubiquitination catalyzed by the deubiquitinating enzymes (Dubs) has recently emerged as another key regulation of the p53-MDM2-MDMX axis [34]. p53 is directly deubiquitinated by several ubiquitin-specific protease (USP) family Dubs, including USP7, USP10, USP29 and USP42, leading to p53 stabilization and activation [35]. USP2 deubiquitinates both MDM2 and MDMX [36, 37] whereas USP4 deubiquitinates ARF-BP1 [38], another ubiquitin ligase for p53, thus indirectly destabilizing p53 and inhibiting its function. We recently found that Otub1, an ovarian tumor (OTU) family member Dub, can stabilize and activate p53 [39]; yet it does so via a non-canonical suppression of the MDM2 cognate ubiquitin-conjugating enzyme (E2), UbcH5, leading to the inhibition of MDM2-mediated p53 ubiquitination [39, 40]. Further, UbcH5 in turn mediates Otub1 monoubiquitination and this monoubiquitination facilitates the Otub1 binding to UbcH5 and likely inhibits ubiquitin chain transfer [40], providing a mechanism underlying the Otub1 inhibition of E2 activity. However, whether Otub1 also regulates MDMX levels and activity is unknown.

Here we report that Otub1 suppresses MDM2-mediated MDMX ubiquitination and degradation. Overexpression of Otub1 markedly stabilized MDMX and induced its levels, whereas knockdown of Otub1 reduced the levels of MDMX. Similar to its regulation of p53, Otub1 stabilizes MDMX independently of its Dub activity. We show that both wild-type Otub1 and its catalytically-inactive mutant (C91S), suppresses MDM2-mediated MDMX ubiquitination in cells and *in vitro*. Interestingly, the stabilized MDMX can localize into mitochondria and mediate mitochondrial apoptotic pathway. Knockdown of MDMX reduced Otub1-induced p53 phosphorylation at S46, which was shown to be critical for p53's mitochondria function, and apoptotic activity. Also, Otub1 promotes UV-irradiation-induced apoptosis, which can be inhibited by MDMX depletion. Together, these results suggest that Otub1 also induces mitochondrial-mediated apoptosis by stabilizing MDMX and relocalizing it to mitochondria to induce mitochondria-mediated apoptosis.

## RESULTS

### Otub1 stabilizes MDMX and increases its levels

**Figure 1:**
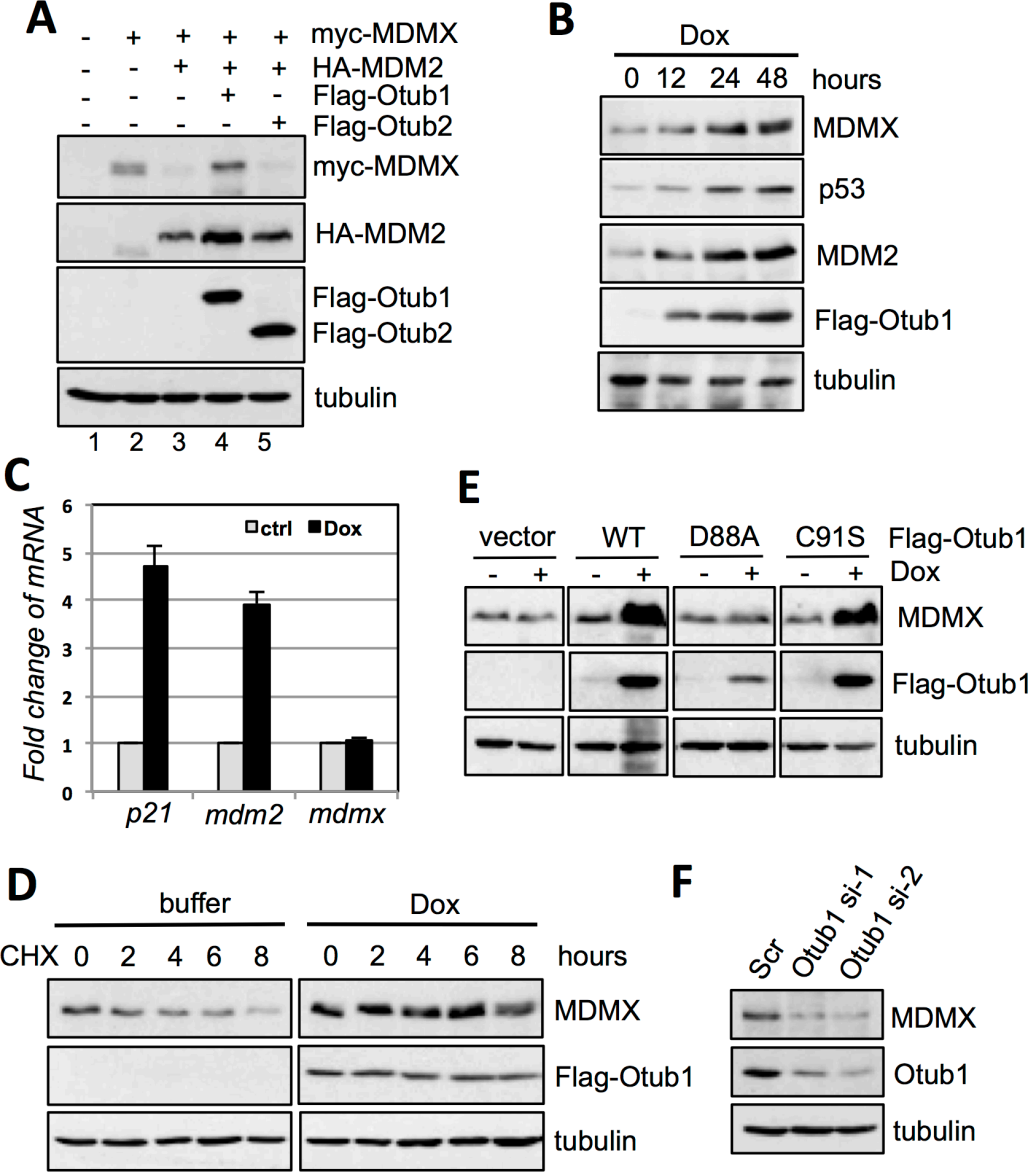
Otub1 stabilizes MDMX and increases its levels (**A**) Otub1, but not Otub2, suppresses MDM2-mediated MDMX degradation. H1299 cells were transfected with the indicated plasmids and assayed by IB. (**B**) Otub1 induces the levels of endogenous MDMX. T-Rex-U2OS-Flag-Otub1 cells were cultured in the presence of 2 ug/ml doxycycline (Dox) for indicated time points, followed by IB. (**C**) Otub1 does not induce the levels of MDMX mRNA. T-Rex-U2OS-Flag-Otub1 cells were cultured in the presence or absence of 2 μg/ml Dox for 24 hours, followed by RT-qPCR detection of the relative expression of *p21*, *mdm2* and *MDMX* mRNA normalized with *GAPDH* mRNA. (**D**) Otub1 stabilizes MDMX. T-Rex-U2OS-Flag-Otub1 cells were cultured in the presence or absence of 2 μg/ml Dox for 24 hours. Then the cells were cultured in the presence of cyclohexamide (CHX, 50 μg/ml) for the indicated hours, followed by IB. (**E**) Otub1 stabilizes MDMX independently of its Dub activity. T-Rex-U2OS-Flag-Otub1 (wt, C91S, or D88A) cells or the control T-Rex-U2OS cells were cultured without or with 2 μg/ml Dox for 24 hours, followed by IB assays. (**F**) Knockdown of Otub1 reduces the levels of MDMX. U2OS cells were transfected with scrambled or individual siRNA against Otub1 and assayed by IB.

To test whether Otub1 regulates MDMX levels, we first performed transient transfection followed by IB analysis in H1299 cells. As shown in Figure 1A and as expected [32, 33], overexpression of MDM2 markedly reduced the levels of MDMX (compare lane 3 to lane 2). Further expression of Otub1 (lane 4), but not Otub2 (lane 5), completely abolished this reduction (Figure 1A). Doxycycline-induced expression of Otub1 in T-Rex-U2OS-Flag-Otub1 cells also drastically induced the levels of MDMX, as well as p53 and its target MDM2 in a time-dependent manner (Figure 1B). This induction of MDMX is due to protein stabilization, as the levels of MDMX mRNA did not change compared to the marked increase of p21 and MDM2 mRNA levels when Otub1 expression is induced (Figure 1C). Also, induced overexpression of Otub1 drastically prolonged the half-life of MDMX as determined by half-life assays (Figure 1D). These results suggest that overexpression of Otub1 alleviates MDM2-mediated MDMX degradation. The stabilization of MDMX by Otub1 does not depend on Otub1's deubiquitinating enzyme activity, as both wild-type (wt) and the catalytic-inactive C91S mutant are equally able to induce the levels of MDMX (Figure 1E). By contrast, the D88A mutant, which is unable to bind to and suppress UbcH5 and unable to induce p53 [39, 40], also failed to induce MDMX levels (Figure 1E), suggesting that this mutant may cause overall structural changes in the OTU domain that abolish Otub1's activity to suppress E2. Consistently, knockdown of endogenous Otub1 by two different siRNAs significantly reduced the levels of MDMX in cells (Figure 1F). Thus endogenous Otub1 also regulates the MDMX levels.

### Otub1 directly suppresses MDM2-mediated MDMX ubiquitination in cells and *in vitro*

**Figure 2:**
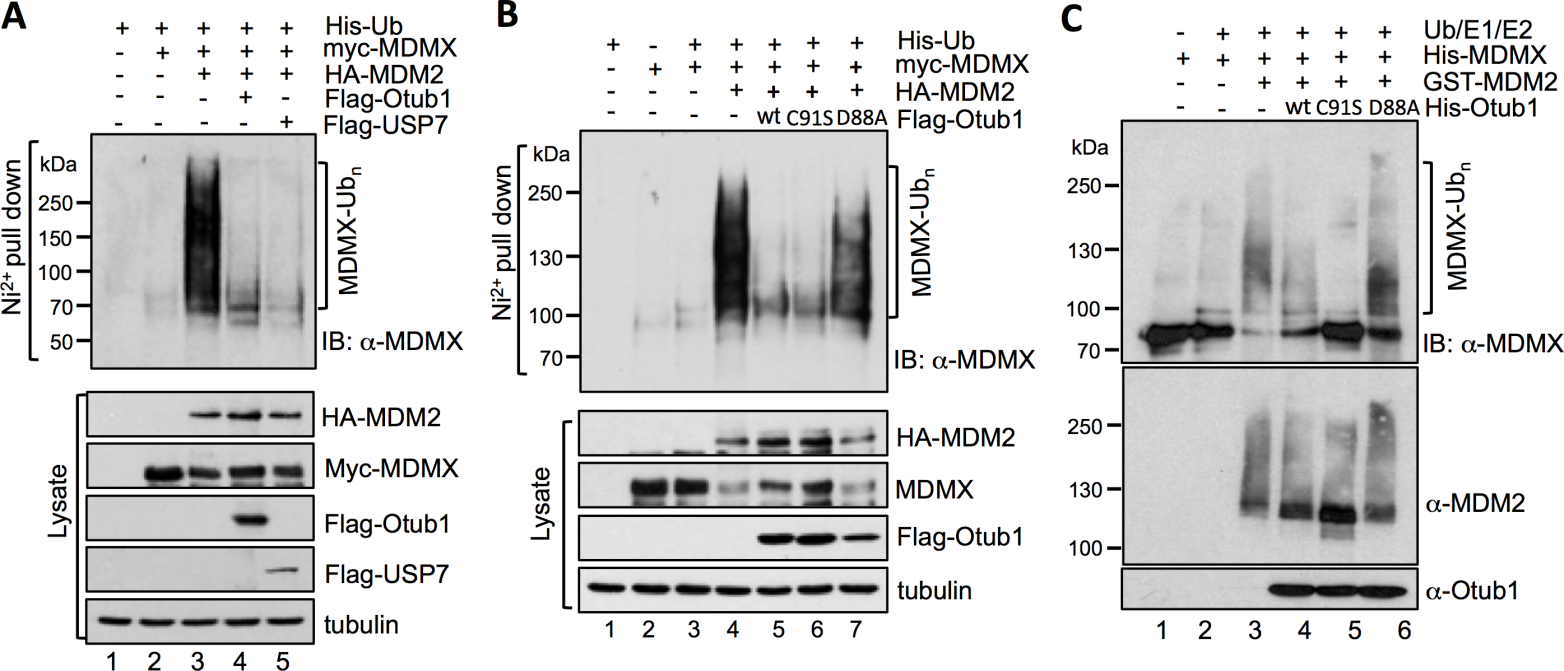
Otub1 suppresses MDM2-mediated MDMX ubiquitination in cells and *in vitro* (**A**) (**B**) wt Otub1 and its C91S mutant, but not the D88A mutant, suppress MDM2-mediated p53 ubiquitination in cells. H1299 cells transfected with different combination of plasmids encoding His-Ub, myc-MDMX, HA-MDM2 in the absence or presence of Flag-Otub1, Flag-USP7 (A), or Flag-Otub1 (wt, C91S, or D88A mutant) (B) were subjected to Ni^2+^-NTA pulldown under denaturing conditions, followed by IB with anti-MDMX to detect the ubiquitinated species of MDMX (top panels). The protein expression is shown in bottom panels. (**C**) wt Otub1 and its C91S mutant, but not the D88A mutant, suppress MDM2-mediated MDMX ubiquitination *in vitro*. The *in vitro* ubiquitination reactions were performed in a reaction mixture containing recombinant Ub, E1, E2 (UbcH5), His-MDMX, GST-MDM2 in the absence or presence of His-Otub1 or its mutants (C91S or D88A), followed by IB with anti-MDMX to detect MDM2-mediated MDMX ubiquitination *in vitro*.

To understand how Otub1 stabilizes MDMX, we tested whether Otub1 suppresses MDM2-mediated MDMX ubiquitination, similar to the suppression of p53 ubiquitination [39, 40]. We first performed *in vivo* ubiquitination assays in H1299 cells using Ni^2+^-NTA purification method. As shown in Figure 2A and expected [32, 33], the ubiquitinated species of MDMX were drastically increased by the overexpression of MDM2 (compare lane 3 to lane 2, top panel). Similar to the role of USP7 as reported (lane 5) [21], further expression of Otub1 markedly reduced MDM2-mediated MDMX ubiquitination (lane 4). This effect does not depend on the Dub activity of Otub1, as overexpression of the C91S mutant also drastically reduced the levels of MDM2-mediated MDMX ubiquitination (Figure 2B), whereas mutating D88 to Ala (D88A) abolished Otub1's activity to suppress MDM2-mediated MDMX ubiquitination (Figure 2B). To examine whether Otub1 directly suppresses MDM2-mediated ubiquitination of MDMX, recombinant His-Otub1 proteins were incubated with recombinant His-MDMX, GST-MDM2, UbE1, UbcH5 (E2), Ub and ATP in the *in vitro* reactions. As shown in Figure 2C, purified wt Otub1 markedly reduced the levels of MDM2-mediated MDMX ubiquitination *in vitro* (compare lane 4 to lane 3). Also, the recombinant His-Otub1^C91S^ (lane 5), but not the His-Otub1^D88A^ (lane 6), mutant also suppressed the MDM2-mediated MDMX ubiquitination. Thus, Otub1 directly suppresses MDM2-mediated MDMX ubiquitination independently of its canonical Dub catalytic activity.

### Otub1-stabilized MDMX localizes to the mitochondria

**Figure 3:**
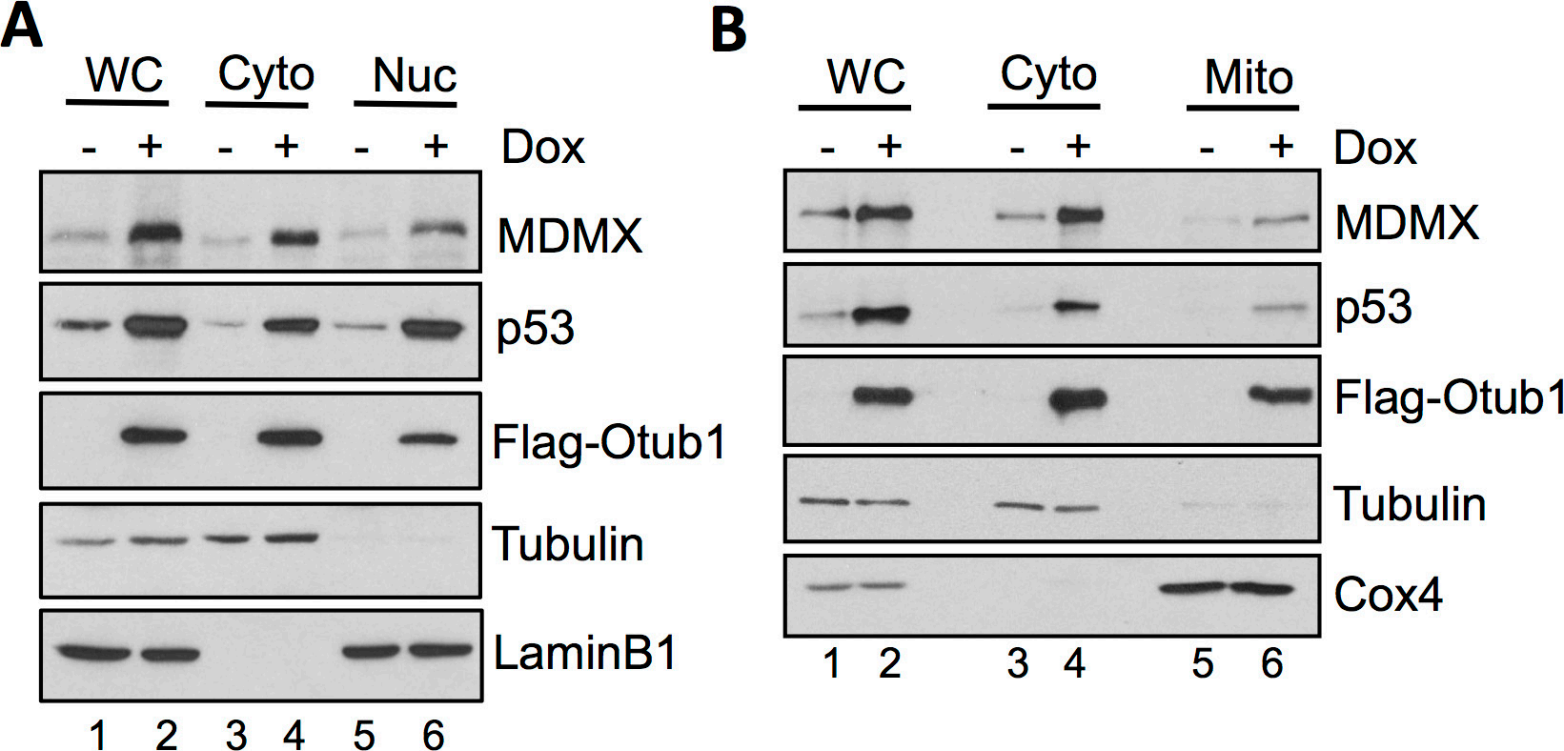
Otub1-stabilized MDMX relocalizes to the cytosol and the mitochondria (**A**) Cell fractionation assays were performed in T-Rex-U2OS-Flag-Otub1 cells treated without or with 2 ug/ml Dox for 24 hour. The cells were fractionated into cytoplasmic (Cyto) and the nuclear (Nuc) fractions, followed by IB with indicated antibodies. (**B**) Otub1-stabilized MDMX relocalizes to the mitochondria. The cytoplasmic fraction was further separated into the cytosol and mitochondria (Mito) fractions by further centrifugation and assayed by IB using the indicated antibodies. WC: whole cell lysates. Tubulin, lamin B1 and Cox4 were used as the cytoplasm, nucleus and mitochondria markers, respectively.

As MDMX assists MDM2 to degrade p53, thus inhibiting p53 function [26–31] while Otub1 expression stabilizes p53 [39], the Otub1-stabilized MDMX may be excluded from suppressing p53. We therefore examined the cellular localization of MDMX upon Otub1 expression. As shown in Figure 3A, cell fractionation assays revealed that Otub1 expression not only significantly induced the nuclear levels of p53 and MDMX, but also induced the levels of cytoplasmic MDMX and p53 (lane 4, Figure 3A). Of note, unlike p53, most of which was accumulated in the nucleus, the majority of MDMX was accumulated in the cytoplasm. These results suggest that Otub1 increases the cytoplasmic levels of both MDMX and p53. As p53 can directly induce mitochondrial apoptosis pathway [4–7] and it has recently been shown that MDMX also possesses pro-apoptotic function in mitochondria [41], we then asked whether MDMX relocalizes to the mitochondria upon Otub1 overexpression. Indeed, mitochondria fractionation assays showed that MDMX (together with p53) was accumulated in the mitochondria fraction (Figure 3B). Thus, Otub1 stabilizes MDMX in the cytosol and promotes MDMX's re-localization to the mitochondria.

### Otub1-stabilized MDMX contributes to p53 S46 phosphorylation and p53-mediated apoptosis

**Figure 4:**
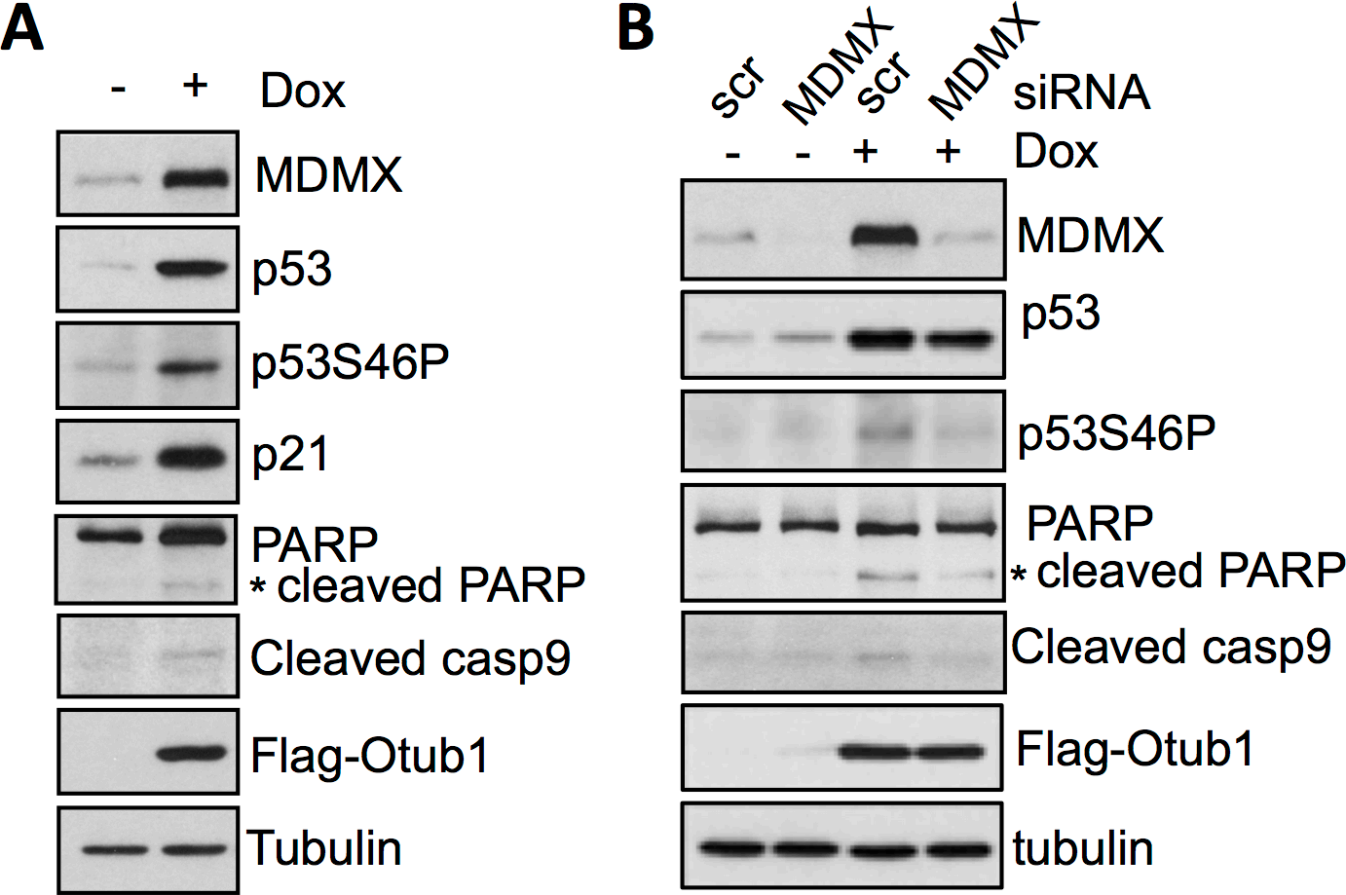
Otub1-stabilized MDMX promotes p53 phosphorylation at S46 and induces apoptosis (**A**) Otub1 expression induces p53-S46P. T-Rex-U2OS-Flag-Otub1 cells treated without or with 2 ug/ml Dox for 24 hours were assayed by IB using indicated antibodies. (**B**) Otub1-induced MDMX participates in the induction of p53S46P. T-Rex-U2OS-Flag-Otub1 cells transfected with scrambled control or MDMX siRNA followed by treatment of cells without or with 2 ug/ml Dox for 24 hours. The cells were assayed by IB using the indicated antibodies.

As described in our previous work, Otub1 stabilizes and activates p53 and can induce apoptosis [39]. It also has been shown that MDMX can promote p53 phosphorylation at S46 (p53-S46P) [41, 42] and this phosphorylation promotes p53 function to induce apoptosis [43, 44]. Indeed, we found that overexpression of Otub1 in U2OS cells induced the levels of p53-S46P (Figure 4A) and caused concomitant induction of cleaved PARP and caspase-9 (Figure 4A), indicative of intrinsic apoptosis. Interestingly, knockdown of MDMX by siRNA significantly attenuated the induction of p53-S46P, cleaved PARP and cleaved caspase-9 by Otub1 overexpression (Figure 4B). Thus, Otub1-stabilized MDMX may be critical for p53 phosphorylation at S46 and play a proapoptotic role in the context of Otub1 overexpression.

### Otub1 promotes UV-induced apoptosis that involves MDMX stabilization

**Figure 5:**
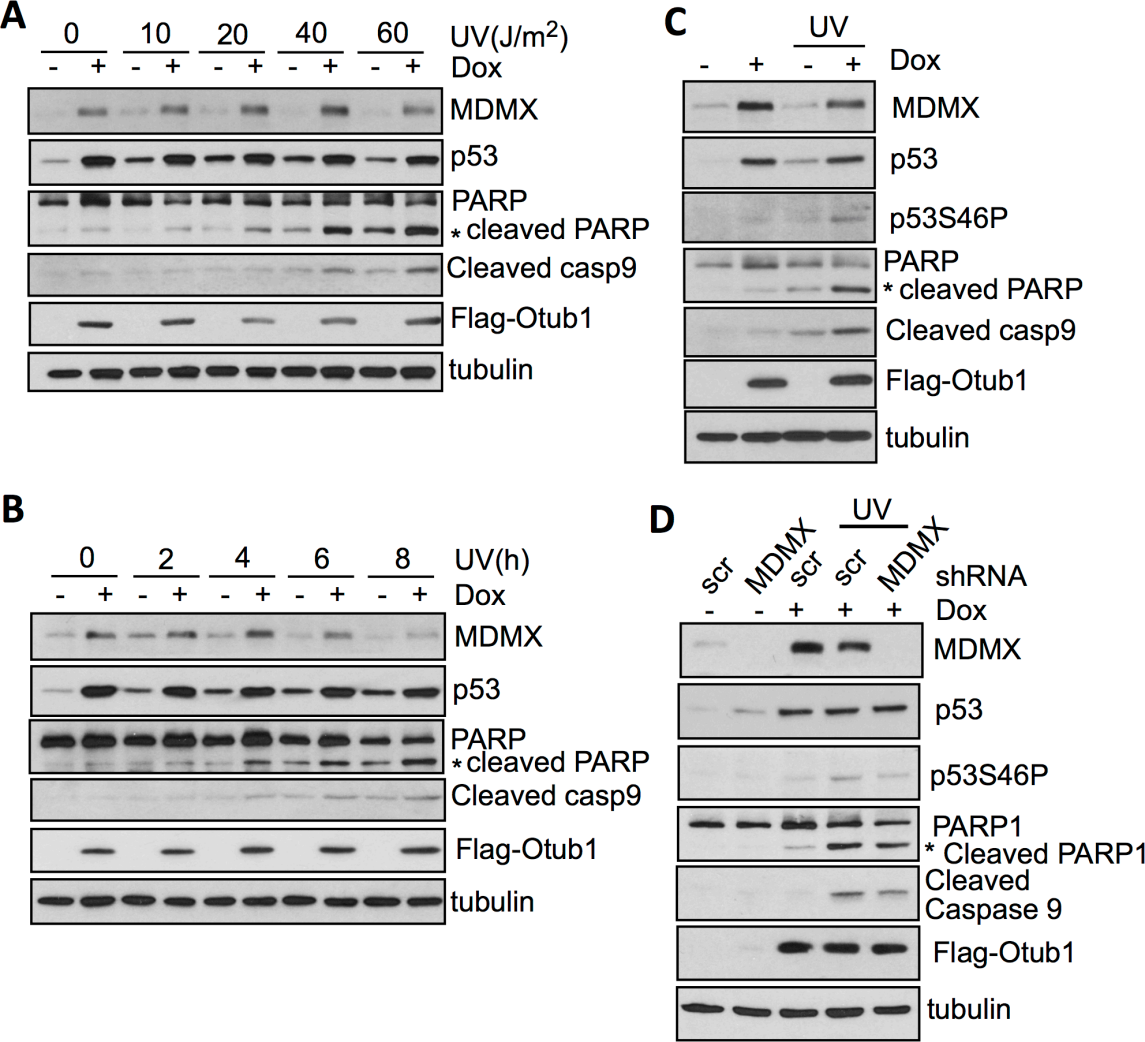
Otub1-stabilized MDMX contributes to p53 phosphorylation at S46 and apoptosis in response to UV irradiation (**A**) (**B**) Otub1 promotes UV irradiation-induced apoptosis. T-Rex-U2OS-Flag-Otub1 cells treated without or with 2 ug/ml Dox for 24 hours were subjected to UV irradiation for 6 hours at different doses (A) or 40 J/m^2^ and harvested at different time points (B). The cells were assayed by IB using indicated antibodies. (**C**) Otub1 promotes p53-S46P following UV irradiation. T-Rex-U2OS-Flag-Otub1 cells were cultured without or with 2 ug/ml Dox for 24 hours, followed by UV irradiation. The cells were assayed by IB using the indicated antibodies. (**D**) Knockdown of MDMX attenuates p53-S46P and induction of apoptosis in response to UV irradiation. T-Rex-U2OS-Flag-Otub1 cells infected with scrambled or MDMX shRNA were cultured without or with 2 ug/ml Dox for 24 hours, followed by UV irradiation (40 J/m^2^) for 6 hours. The cells were then assayed by IB to detect the expression of indicated proteins.

We have previously shown that Otub1 plays a critical role in p53 induction in response to DNA damage such as UV irradiation [39]. Consistent with this notion, induced overexpression of Otub1 significantly promoted UV irradiation-induced apoptosis, as indicated by synergistic induction of the cleaved PARP and Caspase-9 in a time- (Figure 5A) and dose- (Figure 5B) dependent manner. Also, Otub1 expression promoted UV irradiation-induced p53-S46P (Figure 5C). Furthermore, knockdown of MDMX significantly attenuated the induction of p53-S46P, cleaved PARP and Caspase-9 in response to UV irradiation (Figure 5D). Thus, our results support that elevated MDMX can relocalize into the mitochondria, promote p53-S46P, and induce intrinsic apoptosis.

## DISCUSSION

**Figure 6:**
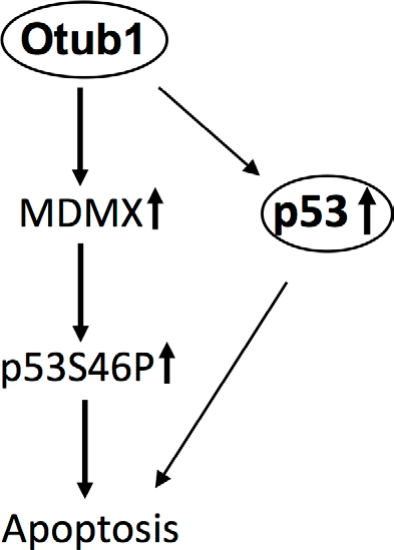
Schematic model for the role of stabilized MDMX by Otub1 in apoptosis In addition to stabilizing p53, Otub1 can also stabilize MDMX, which accumulates in the mitochondria and promotes p53 phosphorylation at S46, resulting in mitochondria-mediated apoptosis.

In this study, we show that Otub1 inhibits MDM2-mediated MDMX ubiquitination, leading to the stabilization of MDMX, its accumulation in mitochondria and the cytosol, p53 S46 phosphorylation, and mitochondria-mediated apoptosis. Knockdown of MDMX impaired both the p53-S46P and the induction of apoptosis induced by Otub1 expression as well as in response to UV irradiation, revealing an important role for MDMX in Otub1-induced and mitochondria-mediated apoptosis (Figure 6). Our finding is also consistent with a previously suggested proapoptotic function of MDMX in mitochondria via promoting p53-S46P and neutralizing anti-apoptotic Bcl-2 family proteins.

As an oncoprotein, the main function of MDMX is to suppress p53 activity and assist MDM2 to ubiquitinate and degrade p53. This inhibitory role is indispensable as deleting p53 rescues the lethal phenotype of the MDMX knockout mice [45–47], similar to that in MDM2^−/−^p53^−/−^ double knockout mice [48, 49]. However, additional evidence suggests that MDMX also possesses intrinsic anti-tumorigenic activity via p53-dependent and p53-independent mechanisms in certain conditions. For example, deleting MDMX increases tumorigenesis in MDM2 transgenic mice, indicating that MDMX can inhibit tumorigenesis when MDM2 is overexpressed [50]. Unlike MDM2^−/−^p53^−/−^ double knockout cells, MDMX^−/−^p53^−/−^ double knockout cells induce multipolar mitosis resulting in increased cell proliferation and spontaneous transformation in MEF cells and increased spontaneous tumorigenesis in mice [51]. Additionally, it has been shown that cisplatin caused cell death requires MDMX-mediated mitochondrial intrinsic apoptosis pathway [52]. Also, overexpression of MDMX has been shown to stabilize p53 and increase p53-dependent cell death in response to stress [53]. Mechanistically, this proapoptotic function occurs in the cytoplasm, as MDMX has been shown to localize into the mitochondria and promote p53-dependent intrinsic apoptotic pathway by facilitating p53 phosphorylation at S46 and the binding of p53 to and neutralizing Bcl-2 [41, 42]. More recently, it has been shown that in response to severe DNA damage, MDMX can dissociate from MDM2 and bind to and stabilize serine-threonine kinase HIPK2 to stimulate p53 phosphorylation at Ser46 [42]. Also, overexpression of MDMX is associated with an increased likelihood of survival in glioma patients [54]. In addition, when highly overexpressed, MDMX can inhibit MDM2-mediated p53 ubiquitination and degradation possibly by competing with MDM2 for binding to p53 and directly suppressing MDM2 autoubiquitination activity [23, 25, 26, 33]. Together, these studies suggest that MDMX, when highly overexpressed, could stabilize p53 and increase p53 cytoplasmic function to mediate intrinsic apoptotic function.

Our observation here supports the proapoptotic function of MDMX when its expression is highly elevated, in this case, by Otub1 overexpression, as MDMX was localized in the mitochondria with concomitant increase of p53-S46P when Otub1 was induced and knockdown of MDMX alleviated the p53-S46P and intrinsic apoptosis. Our finding also extends the function of Otub1 in inducing apoptosis via a novel MDMX-mediated mechanism in the cytoplasm. Thus, it is likely that Otub1 stabilizes p53 and promotes p53′s transcriptional-dependent and transcriptional-independent function with the later mechanism involving MDMX localization in the mitochondria and its role in promoting p53-S46P. The p53-S46P kinase HIPK2 can be activated by UV irradiation-induced DNA damage [43, 44]. p53, once phosphorylated at S46P, can induce key target genes involved in mitochondria-mediated apoptotic pathway. MDMX is also specifically localized in the cytoplasm following UV irradiation [55]. Indeed, we previously showed that Otub1 plays a critical role in p53 stabilization and activation following UV irradiation [56] and in this study we further showed that Otub1 markedly promotes UV-induced p53 activation. Knockdown of MDMX reduced UV-induced p53-S46P and apoptosis upon Otub1 expression. Thus, Otub1 may interplay with MDMX in the cytoplasm to mediate UV-irradiation-induced apoptosis. It is interesting in future studies to test how Otub1 is activated by UV damage to suppress MDM2-mediated MDMX ubiquitination and degradation.

In summary, we have found that Otub1 promotes p53 function by stabilizing MDMX, which in turn contributes to the p53-induced mitochondria-mediated apoptosis pathway. Like the stabilization of p53, the MDMX stabilization also results from the non-canonical role of Otub1 via suppressing ubiquitin E2.

## MATERIALS AND METHODS

### Cell culture, plasmids and antibodies

Human p53-null lung non-small cell carcinoma H1299 and p53-proficient osteosarcoma U2OS cells were cultured in Dulbecco's modified Eagle's medium (DMEM) supplemented with 10% fetal bovine serum (FBS), 50 U/ml penicillin and 0.1 mg/ml streptomycin at 37°C in a 5% CO_2_ humidified atmosphere as previously described [39, 56]. T-Rex-U2OS cells stably transfected with tet-inducible Flag-tagged Otub1 (wild-type, C91S, and D88A) were described [39]. Myc-MDMX and His-MDMX plasmids were cloned by PCR into pcDNA3-Myc and pet24a-His vector, respectively. Flag-Otub1, HA-MDM2, and all other plasmids were previously described [39]. Anti-Flag (M2, Sigma), anti-p53 (DO-1, Santa Cruz), anti-MDM2 (SMP14, Santa Cruz), anti-MDMX (Bethyl Laboratories), anti-p21 (Ab-11, NeoMarkers), anti-cleaved PARP1 (Cell Signaling), anti-cleaved caspase9 (Cell Signaling), anti-Lamin B1 (Abcam), anti-COX4 (Santa Cruz), anti-p53-phospho S46 (Abcam), and anti-V5 (Invitrogen) antibodies were purchased. Rabbit polyclonal anti-Otub1 antibodies were generated as described [39].

### Transfection and immunoblot (IB) analysis

Cells were transfected with plasmids using TransIT^®^-LT1 reagents following the manufacturer's protocol (Mirus Bio Corporation). Cells were harvested at 36–48 hours posttransfection and lysed in lysis buffer consisting of 50 mM Tris-HCl (pH 8.0), 0.5% Nonidet P-40, 1 mM EDTA, 150 mM NaCl, 1 mM phenylmethylsulfonyl fluoride (PMSF), 1 mM DTT, 1 μg/ml pepstatin A, and 1 mM leupeptin. For detection of phosphorylated p53, the phosphatase inhibitor cocktails (Sigma) were added in above lysis buffer. Equal amounts of clear cell lysate were used for IB analysis.

### *In vivo* ubiquitination assay

*In vivo* ubiquitination assay under denaturing conditions was conducted in H1299 cells as previously described [39, 56]. Briefly, cells transfected with indicated plasmids were treated with 40 μM MG132 for 6 h before harvesting. The cells were harvested at 48 h after transfection, and 20% of the cells were used for direct IB and the rest of cells were used for ubiquitination assays under denaturing conditions using Ni^2+^-NTA pulldown. After washing, the bead-bound proteins were analyzed by IB.

### *In vitro* ubiquitination assay

Recombinant His-Otub1 (WT, C91S, and D88A) and His-MDMX proteins were expressed in *E. coli* and purified using Ni^2+^-NTA purification method as previously described [39, 40]. GST-MDM2 protein was expressed in E. coli and affinity purified with glutathione-Sepharose 4B (Amersham Biosciences) and eluted with glutathione. The *in vitro* ubiquitination reactions were assembled in a total of 20 μl reaction mixture containing recombinant UbE1 (0.025 μM, Boston Biochem), UbcH5 (0.4 μM, Boston Biochem), Ub (40 μM, Boston Biochem), 50 mM Tris-HCl (pH 8.0), 5 mM MgCl_2_, 2 mM ATP and 1 mM DTT in the absence or presence of 3.2 μM purified His-Otub1 at 37^°^C for 2 hours, followed by IB as described previously [39, 40].

### RNA interference (RNAi)

The 21-nucleotide siRNA duplexes with a 3′ dTdT overhang were synthesized by Dharmacon Inc (Lafayette, CO). The target sequences for Otub1 are 5′-GACCAGGCCTGACGGCAAC-3′ (siRNA-1), 5′-GCAGACCTCTGTCGCCGAC-3′ (siRNA-2). The control scramble RNA sequence was described [39]. MDMX knockdown was performed using either siRNA (5′-AGATTCAGCTGGTTATTAA-3′ [57] or lentiviral-encoded shRNA (Open Biosystems). Cells were transfected with these siRNA duplexes using SilentFect Lipid Reagent (Bio-Rad) following the manufacturer's protocol or infected with shRNA-encoding lentiviruses as described [56]. The cells were analyzed 48 hours after transfection or infection.

### Reverse transcriptase-Quantitative polymerase chain reaction (RT-qPCR) analysis

Total RNA was isolated from cells using Trizol^®^ reagent following manufacturer's protocol (Invitrogen). Reverse transcriptions were performed as described [39]. Quantitative real-time PCR was performed on an ABI 7300 real-time PCR system (Applied Biosystems) using iTaq^™^ Universal SYBR Green Supermix (Bio-Rad) as described previously [39, 58]. All reactions were carried out in triplicate. The relative gene expression was calculated using the ΔCτ method following the manufacturer's protocol. The primers for *p21*, *mdm2* and *GAPDH* were described [39]. The primers for MDMX are 5′-GCCTTGAGGAAGGATTGGTA-3′ and 5′-TCGACAATCAGGGACATCAT-3′.

### Cell fractionation

T-Rex-U2OS-Flag-Otub1 cells cultured in the presence or absence of doxycycline (2 μg/ml) were resuspended in a hypotonic buffer A (10 mM HEPES pH 7.9, 10 mM KCl, 1.5 mM MgCl2, 0.5 mM DTT) and homogenized. After centrifugation, the supernatant was collected as the cytoplasmic fraction. The pellets were resuspended in buffer C (20 mM HEPES pH 7.9, 420 mM NaCl, 0.2 mM EDTA, 1.5 mM MgCl2, 0.5 mM DTT, 25% Glycerol) and sonicated. The nuclear fraction (supernatant) was collected by centrifugation. For isolating mitochondria, cells were trypsinized and washed twice in phosphate-buffered saline (PBS) and resuspended in ice-cold IB_cells_-1 buffer (225 mM mannitol, 75 mM sucrose, 0.1 mM EGTA, 30 mM Tris-HCl pH7.4, 1 mM DTT and protease inhibitors). After hypotonic swelling on ice, the cells were homogenized in dounce tissue grinder until 80–90% of cell damage was attained. The homogenate was centrifuged twice at 600 g for 5 min at 4°C. The supernatant was collected and centrifuged at 7000 g for 10 min at 4°C. The supernatant was saved as the cytoplasmic fraction, and the mitochondrial pellet was resuspended in IB_cells_-2 buffer (225 mM mannitol, 75 mM sucrose, 30 mM Tris-HCl pH7.4, 1 mM DTT and protease inhibitors), followed by a 10 min centrifugation at 10,000 g. The mitochondrial pellet was lysed in mitochondria lysis buffer (0.5% TritonX-100, 30 mM Tris-HCl pH7.4, 200 mM KCl, 5 mM EDTA, 1 mM DTT and protease inhibitors) and assayed by IB.
